# The impact of an integrated safer use space and safer supply program on non-fatal overdose among emergency shelter residents during a COVID-19 outbreak: a case study

**DOI:** 10.1186/s12954-022-00614-8

**Published:** 2022-03-21

**Authors:** Brendan Lew, Claire Bodkin, Robin Lennox, Timothy O’Shea, Gillian Wiwcharuk, Suzanne Turner

**Affiliations:** 1 Department of Family Medicine, McMaster University, 100 Main St West, Hamilton, ON L8P 1H6 Canada; 2 Department of Health Research Methods, Evidence and Impact, McMaster University, 1280 Main St West, Hamilton, ON L8S 4K1 Canada; 3 Department of Medicine, McMaster University, 1200 Main St West, Hamilton, ON L8N 3Z5 Canada

**Keywords:** Case study, Substance use, Homeless shelters, Overdose, Controlled substances

## Abstract

**Background:**

Opioid-related harms, including fatal and non-fatal overdoses, rose dramatically during the COVID-19 pandemic and presented unique challenges during outbreaks in congregate settings such as shelters. People who are deprived of permanent housing have a high prevalence of substance use and substance use disorders, and need nimble, rapid, and portable harm reduction interventions to address the harms of criminalized substance use in an evidence-based manner.

**Case study:**

In February 2021, a COVID-19 outbreak was declared at an emergency men’s shelter in Hamilton, Ontario, Canada. Building on pre-existing relationships, community and hospital-based addictions medicine providers and a local harm reduction group collaborated to establish a shelter-based opioid agonist treatment and safer supply program, and a volunteer run safer drug use space that also distributed harm reduction supplies. In the 4 weeks preceding the program, the rate of non-fatal overdoses was 0.93 per 100 nights of shelter bed occupancy. During the 26 days of program operation, there were no overdoses in the safer use space and the rate of non-fatal overdoses in the shelter was 0.17 per 100 nights of shelter bed occupancy. The odds ratio of non-fatal overdose pre-intervention to during intervention was 5.5 (95% CI 1.63–18.55, *p* = 0.0059). We were not able to evaluate the impact of providing harm reduction supplies and did not evaluate the impact of the program on facilitating adherence to public health isolation and quarantine orders. The program ended as the outbreak waned, as per the direction from the shelter operator.

**Conclusions:**

There was a significant reduction in the non-fatal overdose rate after the safer drug use and safer supply harm reduction program was introduced. Pre-existing relationships between shelter providers, harm reduction groups, and healthcare providers were critical to implementing the program. This is a promising approach to reducing harms from the criminalization of substance use in congregate settings, particularly in populations with a higher prevalence of substance use and substance use disorders.

## Background

The coronavirus disease 2019 (COVID-19) pandemic has disproportionately impacted people living in congregate settings including emergency shelters [1]. Shelters are high risk settings for COVID-19 outbreak due to the crowded and communal environment, as well as the higher rates of comorbidities among people deprived of housing. Homeless individuals in one Canadian study were disproportionately impacted by overdoses during reopening compared to those individuals with stable housing [2]. Shelter-in-place orders, in the USA, were associated with a higher risk of emergency room visits for overdose [3]. Co-exposure to stimulants and synthetic fentanyls continues to drive overdose deaths before and during the pandemic [4, 5]. People who use drugs who are required to isolate or quarantine due to COVID-19 may be pushed to consume unfamiliar drugs in unfamiliar settings, raising risk of overdose [6].

A recent systematic review demonstrated that supervised consumption facilities reduce overdose rates and improve access to healthcare. Also, pharmacological interventions including opioid agonist therapy can reduce harms in this population [7]. Access to harm reduction services including off-premises supervised consumption sites and harm reduction supplies may be interrupted due to infection prevention and control measures during a COVID-19 outbreak [8]. In the context of an increasingly toxic drug supply, this increases the risk of fatal and nonfatal overdose and other negative health outcomes related to substance use [8]. The Canadian Research Initiative in Substance Misuse (CRISM) has issued guidance that supervised consumption models and providing pharmaceutical grade medications to people with ongoing opioid use can be operated amidst the COVID-19 pandemic [6]. In this scenario, oral pharmaceutical-grade tablets are prescribed in-risk mitigation paradigm. Oral formulations are dissolved and injected by the patient. There are harms associated with oral formulations that are injected secondary to the binders, emulsifiers, colorings and lubricants present [9]. The prescription of oral hydromorphone tablets, for patients to crush and inject, as a harm-reduction strategy is a widely presented recommendation in Canada to reduce harms from the poisoned, illicit opioid supply.

Physicians can play an important role in the provision of healthcare to people deprived of housing or utilizing shelter resources and often participate in tailored care for these individuals [10]. This can include prescribing of opioid agonist therapy or other pharmaceutical grade opioids, assessment for COVID-19 infection, and management of comorbidities. Prescribing unwitnessed doses of pharmaceutical opioids to individuals for whom traditional opioid agonist therapy may not be sufficient treatment, or where they may not be interested or able to engage in opioid agonist therapy at all, may help to mitigate the harms related to the toxic drug supply that are heightened during a COVID-19 outbreak in a shelter setting [11]. This is also known as safer supply or risk mitigation prescribing [11].

We aim to describe the implementation and impact of an emergency safer use space (SUS) in a shelter setting in COVID-19 outbreak, including provision of harm reduction services, initiation of opioid agonist therapy, and safer supply prescribing using hydromorphone tablets.

## Case presentation

A COVID-19 outbreak was declared in a 92 bed emergency adult men’s shelter for people deprived of housing in Hamilton, ON, with 63 cases attributed to the outbreak.

This shelter provides temporary housing for men in the lowest income quartile, in addition to meals, case management and spiritual support. National data estimate that approximately 30% of shelter-users engage in substance use at any one time [12]. This shelter had an abstinence-based approach to substance use prior to and after the COVID-19 outbreak. Common health conditions in the shelter system include seizures, chronic obstructive pulmonary disease, arthritis, hypertension, diabetes, and anemia. Unfortunately, these conditions are often undetected or poorly controlled for long periods of time and contribute to a high mortality rate [13]. A shelter-embedded health clinic provided primary care by a dedicated staff of family physicians and internists with expertise in harm reduction, addiction medicine as well as primary inner-city health care. Several of these physicians held other roles as hospital and community-based physicians. The shelter-associated physicians recognized that many residents would be at risk for heightened harms due to the toxic drug supply and self-isolation requirements and may also have difficulty adhering to public health restrictions during the outbreak without additional support. A partnership formed between the Hamilton Social Medicine Response Team (HAMSMaRT), Keeping Six Hamilton Harm Reduction Action League, and the inpatient addiction medicine service (iAMS) to address this need. HAMSMaRT is a grassroots organization that strives to provide excellence in clinical care to individuals who have difficulty accessing the traditional medical system. Keeping Six is a community-based organization that defends the rights, dignity and humanity of people who use drugs. The iAMS is a multidisciplinary team of physicians who provide care to people who use drugs in two academic hospitals. The partnership’s primary goal was to provide substance-related support to the shelter residents during the outbreak, with a focus on reducing harm from the toxic drug supply and enabling residents to adhere to public health orders. Networks from both HAMSMaRT and Keeping Six were rapidly engaged to provide on-site volunteer and paid peer support.

**Fig. 1:**
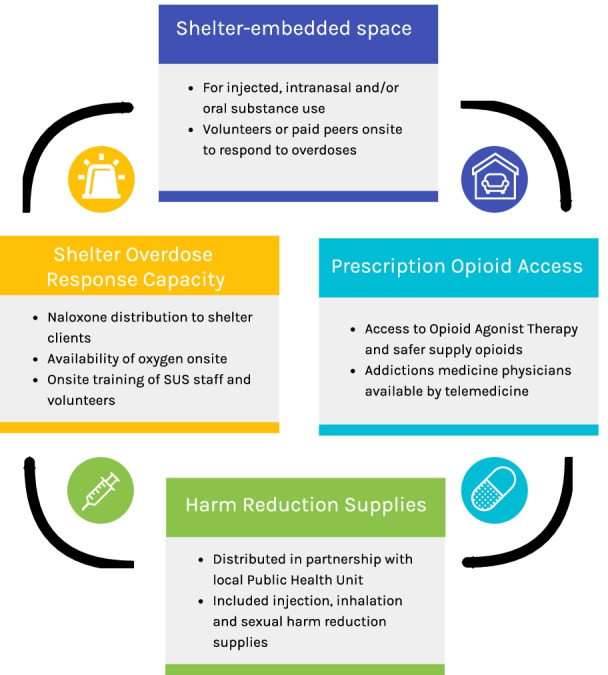
Four Components of the integrated SUS and safer supply program

The integrated SUS and safer supply program had four main components (Fig. 1).

**Fig. 2:**
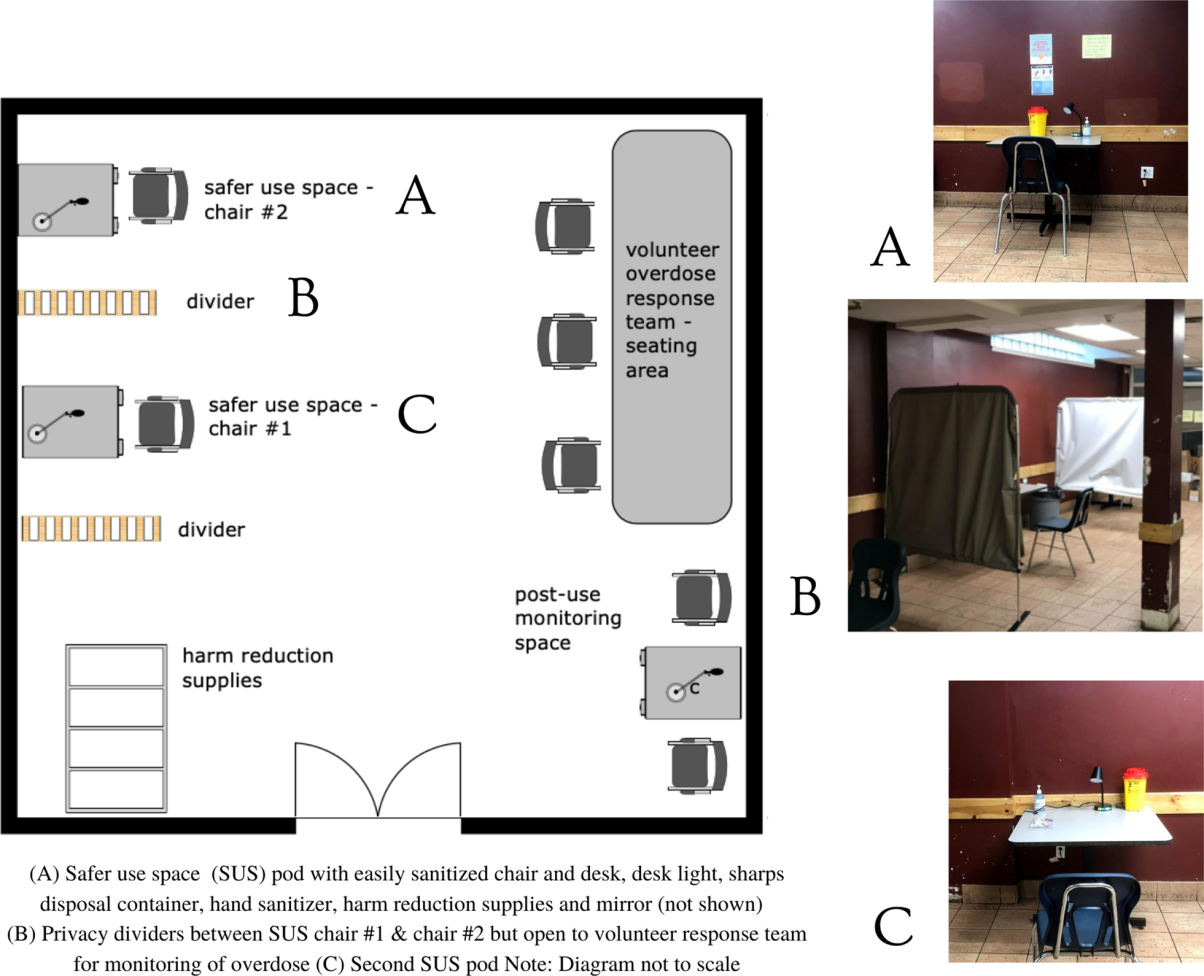
Safer use space (SUS) layout

The first component was a shelter-embedded space where volunteers could observe shelter residents using prescribed safer supply (hydromorphone tablets in this case) via injection, intranasal and/or oral routes. The SUS did not have appropriate ventilation to allow residents to use smoked or inhaled substances. The SUS was able to accommodate two shelter residents at a time with two volunteers or paid peers observing who were trained in overdose response (Fig. 2).

It was open for 10–16 h/day. All volunteers had access to an iAMS physician via telephone for the duration of their shift. Given the rapid implementation time, applying for a Section 56 federal exemption for a supervised consumption site (SCS) or Urgent Public Health Need Site was not feasible. SCS status is required under the criminal code to allow attendees to use controlled substances under observation. As a result, only prescribed hydromorphone was intended to be consumed in the SUS. During the program’s operation, there were over 120 visits by shelter-residents to the SUS to use substances under observation.

The second component was to prescribe opioid agonist treatment (OAT) and opioids within a safer supply paradigm. On-call physicians from the iAMS provided in-person or phone assessments for opioid agonist therapy with buprenorphine/naloxone, methadone, or slow-release oral morphine (SROM). In addition, hydromorphone tablets were prescribed as safer supply, to be used in place of fentanyl or other non-prescribed opioids. While this is an off-label use of hydromorphone, this prescribing paradigm was consistent with British Columbia Centre on Substance Use COVID-19 risk mitigation guidelines [6]. Five unique individuals were prescribed hydromorphone tablets and a long-acting opioid (methadone or SROM or buprenorphine/naloxone). This number does not reflect residents that were previously prescribed on an opioid agonist. Participants were encouraged but not required to use the SUS to consume their safer supply prescription. In the 26 days the SUS was operating, there were 129 visits and corresponded to approximately 5 visits per day.

The third component was a partnership with the local public health unit to distribute harm reduction supplies from the SUS (Table 1). Shelter residents were able to access safer injection supplies, safe inhalation supplies, and condoms, regardless of their use of the SUS.

**Table 1:** Total number of harm reduction supplies distributed

Item	Number distributed
Number of people accessing harm reduction supplies	125
Methamphetamine pipes	87
Inhalation kits (2 stems per kit)	19
Individual needle/syringe	151
2 hit kit (2 × needles, 2 × syringes, 2 × sterile water, 2 × cookers, 2 × alcohol swabs, tourniquets, Vitamin C, matches)	107
10 hit kit ((10 × needles, 10 × syringes, 10 × sterile water, 10 × cookers, 10 × alcohol swabs, tourniquets, Vitamin C, matches)	12
Foil	20
Sharps containers	3
Condoms	10
Lubricant	10

These totals did not include the supplies utilized by patients while accessing the space for injection and intranasal use on site

The fourth component was increasing overdose response capacity within the shelter. A local pharmacy facilitated distribution of naloxone kits to shelter residents, and for overdose response in the SUS and on shelter premises. A local paramedic group provided oxygen for use in the SUS. A series of training videos were developed for the SUS volunteers including overdose response, use of oxygen, and proper use of personal protective equipment (PPE). Significant efforts were made to provide in-person training, including overdose response simulation, for SUS volunteers at the beginning of each shift during the first week. An electronic training manual was provided to all SUS volunteers, with a printed copy located in the SUS.

Data were collected regarding non-fatal overdoses before and during the intervention and the rates of overdose (Table 2). Overdoses were defined as: (1) episodes of respiratory or cardiac depression requiring volunteer intervention in the SUS or (2) episodes of respiratory, cardiac depression or loss of consciousness identified by shelter staff as requiring intervention and thought to be secondary to substance exposure. There were no fatal overdoses during either time-period. In the 4 weeks before the COVID-19 outbreak, there were 20 non-fatal overdoses attended to by shelter staff. This is a rate of 0.93 non-fatal overdoses per 100 nights of shelter bed occupancy. During the 26 days the SUS and safe supply program operated, there were no overdoses in the SUS. There was a total of 3 non-fatal overdoses that were responded to in the shelter resulting in a rate of 0.17 non-fatal overdoses per 100 nights of shelter bed occupancy. The odds ratio of non-fatal overdose pre intervention to during intervention was 5.5 (95% CI 1.63–18.55, *p* = 0.0059). The program ended as the outbreak waned, as per the direction from the shelter operator.

**Table 2:** Non-fatal overdoses before and during the intervention

	28 days prior to SUS operation (Jan 27–Feb 21 2021)	During SUS operation Feb 22–March 19 2021 (26 days)
Total nights of shelter provided (sum of occupied beds per night for the period)	2154	1778
Number of non-fatal overdoses	20	3^a^
Rate (non-fatal overdoses per 100 nights of shelter bed occupancy)	0.93	0.17

^a^None of these occurred in the SUS; they were on site at the shelter outside of the SUS

## Discussion and conclusions

The integrated safer drug use and safer supply harm reduction program introduction was associated with a significant reduction in the non-fatal overdose rate. It helped to mitigate harms related to the reduction and restriction of services including supervised consumption facilities, harm reduction supplies, and substance use care. Fuertes et al. demonstrated that mobile drug consumption rooms were highly utilized in Lisbon’s response to the COVID-19 shelter-response and mobile consumption may be an area of further research needed when addressing overdose response in Canadian shelters [14]. Similarly, in South Africa’s response to homelessness in a large urban center, primary care practitioners prioritized the provision of methadone to reduce withdrawal [15]. Given that methadone and buprenorphine treatment are readily available in the urban shelter system in this study location and overdoses are common despite this availability, it was felt that OAT alone (without risk-mitigation hydromorphone tablet prescribing or without the ability to offer direct observation of substance use) would not be effective in preventing overdoses. In Tyndall’s review of safer opioid prescribing in the context of COVID-19, he discusses the challenges associated with finding physicians willing to prescribe pharmaceutical-grade opioids to PWUD [11]. This study demonstrates that when prescription opioids are offered to patients in a congregate setting, patients are willing to engage in this form of treatment even when the expectation is that prescription opioids are consumed in a monitored setting such as a SUS.

It underscores the responsibility of healthcare providers to address the needs of shelter residents and people who use drugs during the COVID-19 pandemic. It also highlights the value of collaboration among shelter providers, harm reduction groups, and healthcare providers in responding to public health emergencies.

The ability of this program to distribute harm reduction supplies (Table 1) during a COVID-19 shelter outbreak, particularly when other harm-reduction services had greatly reduced hours or were closed, provides a model for future COVID-19 outbreak response initiatives. Notably, more than 100 methamphetamine pipes or safer inhalation kits were distributed by the SUS volunteers during the 4-week period. Further, in addition to harm reduction injection supplies reducing risk of skin and soft tissue and blood-borne infections, safer inhalation kits have the potential to reduce risk of transmission of respiratory illness such as COVID-19 related to sharing of equipment [16]. This demonstrates a significant unmet need for safe consumption spaces and risk mitigation strategies for people who use inhaled substances and may have helped reduce COVID-19 spread by limiting the sharing of paraphernalia for inhaled substances.

The SUS model was initially limited by the lack of trust that shelter residents felt about accessing the space. The shelter itself had an abstinence-based policy towards substances. For example, shelter residents could be restricted from accessing the shelter if found to have drug use paraphernalia on-site. As such, the harm reduction approach paired with the implementation of the SUS was in direct conflict with the existing shelter policies that residents were familiar with. The rapid mobilization of a safer use space and encouragement to access harm reduction services within this context was confusing for clients, and it took time for them to gain the necessary trust to access these services.

The program evaluation had several limitations. We did not collect any identifiers for shelter residents using the SUS and as a result we do not have data demonstrating the number of unique residents who accessed the SUS or the frequency with which residents accessed the site. Because the program was established so rapidly there was no formal evaluation framework, and we had no way to identify or contact SUS clients after the site closed to obtain their feedback. As this was not a controlled experimental study, it is not possible to conclude that the combined program intervention caused the reduction in overdoses or that there were no other confounding variables that led to the reduction in overdoses. However, an integrated safer drug use and safer supply harm reduction program is a promising approach to reducing substance-related harms in congregate settings, particularly during a pandemic-related outbreak. More rigorous studies are needed to establish the impact of an integrated program, such as was established during this COVID-19 outbreak. Future studies would be helpful to establish the direct impact of this type of intervention and reduce confounders that may have been contributing to the overdose reduction seen in this program description. Future interventions should address the needs of individuals who inhale substances. To establish trust and have a sustained impact, long-term interventions rather than short-term, episodic interventions should be prioritized.

## Data Availability

The datasets used and analyzed during the current study are available from the corresponding author on reasonable request.

## Abbreviations

CRISM: Canadian Research Initiative in Substance Misuse
HAMSMaRT: Hamilton Social Medicine Response Team
iAMS: Inpatient Addictions Medicine Service
OAT: Opioid agonist treatment
PPE: Personal protective equipment
SCS: Supervised consumption site
SROM: Slow release oral morphine
SUS: Safer use space
